# Multivariate permutation test to compare survival curves for matched data

**DOI:** 10.1186/1471-2288-13-16

**Published:** 2013-02-11

**Authors:** Stefania Galimberti, Maria Grazia Valsecchi

**Affiliations:** 1 Center of Biostatistics for Clinical Epidemiology, Department of Health Sciences, University of Milano-Bicocca, 20900, Monza, Italy

**Keywords:** Highly stratified data, Matched survival data, Multiple matching, Multivariate permutation tests

## Abstract

**Background:**

In the absence of randomization, the comparison of an experimental treatment with respect to the standard may be done based on a matched design. When there is a limited set of cases receiving the experimental treatment, matching of a proper set of controls in a non fixed proportion is convenient.

**Methods:**

In order to deal with the highly stratified survival data generated by multiple matching, we extend the multivariate permutation testing approach, since standard nonparametric methods for the comparison of survival curves cannot be applied in this setting.

**Results:**

We demonstrate the validity of the proposed method with simulations, and we illustrate its application to data from an observational study for the comparison of bone marrow transplantation and chemotherapy in the treatment of paediatric leukaemia.

**Conclusions:**

The use of the multivariate permutation testing approach is recommended in the highly stratified context of survival matched data, especially when the proportional hazards assumption does not hold.

## Background

In clinical trials with right-censored failure time outcome, inference often focuses on the comparison of survival curves. When data from observational studies are used to explore the role of different treatments, the main problem is to limit the biases due to the lack of randomization. Matching on relevant baseline features can be used in order to increase the comparability between subjects treated with an experimental therapy and those receiving standard treatment. In settings where matching is done with a variable number of controls (multiple matching), highly stratified data are produced, with strata containing a few, possibly censored, observations. For this reason, the statistical comparison of survival in the two groups cannot be directly addressed by means of the usual nonparametric procedures, such as the log-rank test. The stratified version of these tests, which should account for matching, is inefficient when the number of strata increases and the stratum size is small [1]. Furthermore, these methods are less sensitive when proportional hazard is not satisfied and this might often be the case, especially in the clinical setting that motivated this paper, i.e. the comparison of bone marrow transplantation and chemotherapy in the treatment of leukemia [2,3].

The comparison of the survival curves for highly stratified data due to non-paired matching was addressed by Galimberti et al. [2], who proposed a weighted Kaplan-Meier estimator for the controls and a nonparametric permutation test on the survival difference at one pre-fixed time point. The aim of this work is to extend the comparison of survival at multiple time points, using the multivariate permutation approach originally introduced by Pesarin [4]. In the proposed procedure, differently from Pesarin and Salmaso [5], a desirable feature is that there is no need to resort to the missing data framework in order to deal with censoring.

Our method is compared with the stratified log-rank test, the modified log-rank test for highly stratified data of Schoenfeld and Tsiatis [1] and the Cox model with a sandwich robust standard error for the treatment effect [6,7]. There were no natural competitors among other permutation tests on survival proposed in the literature [8-10].

The paper is organized as follows. In the next section, we show how the multivariate permutation approach can be extended to the survival matched data setting. We investigate the performance of the proposed test through simulation studies that are presented in the Simulation section and we illustrate the method, using data from the motivating study on childhood leukemia [11], in the Application section. Finally, we provide a discussion and concluding remarks.

## Methods

### Multivariate permutation tests for survival matched data

In the context of non randomized studies, especially in rare diseases, where only selected patients undergo experimental therapies, matching is an approach to identify a proper set of controls for an unbiased comparison. This was the case of a multicenter study conducted in Italy on the role of allogeneic Bone Marrow Transplantation (BMT) from matched sibling donors in children with acute lymphoblastic leukaemia (ALL) who, by presenting features, have a dismal prognosis [11]. Matching produced a stratum for each transplanted patient, that included as many controls as available, in order to recover the maximum amount of information in this rare subgroup.

More formally, this setting presents finely stratified data: each of the k strata (j = 1,…,k) has 1+m_j_ subjects, where the first is the case belonging to the experimental arm (group 1) and the remaining m_j_ are the matched subjects in the control arm (group 2).

The available data refer to the potential survival and censoring times. We assume that, conditionally on the observed j-th stratum, the survival times of the m_j_ controls are i.i.d., but we cannot exclude, due to matching, some dependence between the case and the corresponding controls. Moreover, the usual assumption that censoring is independent of the failure mechanism and of the treatment must hold within stratum. Across strata, both cases and controls are independent, but not necessarily identically distributed. Finally, under the null hypothesis, the distribution of the survival times of the case and the matched controls in each stratum is invariant under permutations.

The multidimensional permutation approach introduced by Pesarin is here adapted to the context of survival analysis with matched data for the comparison of the marginal survival distributions of the two groups, S_1_(t) and S_2_(t). The procedure is based on a nonparametric combination of multiple, possibly dependent, univariate permutation tests. It relies on the following main steps: reformulate the inferential question of interest into a set of sub-questions, set up the partial tests and set up the form for their combination [4].

Firstly, the null and the alternative hypothesis are decomposed into a finite set of q sub-hypotheses H_0i_ and H_1i_ (i=1,…,q) with the property that, if H_0_ is true, all the H_0i_ are jointly true, while if H_1_ is true, at least one sub-hypothesis is true. The standard hypotheses on the survival distributions are thus rephrased into q sub-hypotheses H_0i_, H_1i_ (i=1,…,q) so that:

H_0_: S_1_(t)=S_2_(t) becomes H_0_:$\overset{\text{q}}{\underset{\text{i}=1}{\cap }}{\text{H}}_{0\text{i}}$with H_0i_: S_1_(t_i_)=S_2_(t_i_),H_1_: S_1_(t)≠S_2_(t) becomes H_1_:$\overset{\text{q}}{\underset{\text{i}=1}{\cup }}{\text{H}}_{1\text{i}}$ with H_1i_: S_1_(t_i_)≠ S_2_(t_i_).

Appropriate partial tests T_i_^1^ are then performed separately on each q sub-hypothesis H_0i_ (i=1,…,q). We considered two versions of test statistics that are marginally unbiased and consistent, as required for the validity of the procedure [4]. They are based on the distance of the two survival curves or on their complementary log-log transformation [12] estimated at q different times points (t_1_,.,t_i_,.,t_q_):

$${\mathrm{TS}}_{i}^{1}={\hat{S}}_{1}\left(t_{i}\right)-{\hat{S}}_{2}^{w}\left(t_{i}\right)\left(i=1,\ldots ,q\right)$$

TLS_i_^1^=log{-log[${\hat{S}}_{1}$(t_i_)]}-log{-log[${\hat{S}}_{2}^{w}$(t_i_)]}

where ${\hat{S}}_{1}\left(.\right)$ is the usual Kaplan-Meier estimate of the survival distribution in group 1, while ${\hat{S}}_{2}^{w}\left(.\right)$ is a weighted version, with stratum specific weights used in group 2 to account for the variable number of subjects in each stratum [2]. If we indicate with d_j_(u) and r_j_(u) the number of events and the number of subjects at risk at time u in stratum j, and with w_j_ =1/m_j_ the weight applied to the m_j_ controls of the stratum, the formula for ${\hat{S}}_{2}^{w}\left(.\right)$ is:

$${\hat{S}}_{2}^{w}\left(t\right)=\prod_{u:u\leq t}\left(1-\frac{\sum_{j=1}^{k}w_{j}\;d_{j}\left(u\right)}{\sum_{j=1}^{k}w_{j}\;r_{j}\left(u\right)}\right)$$

which can also have a product integral representation for continuous time variables. This estimator has an intuitive appeal because it can be viewed as a Kaplan-Meier estimator computed on k components, one for each stratum, in which all the m_j_ controls are given the same weight. The imbalance in prognostic factors induced by multiple matching is adjusted giving less weight to individuals from strata that are over-represented and vice versa. The idea behind this estimator is to standardize the survival curve of controls, using the population of cases as reference. The weighted Kaplan-Meier estimator ${\hat{S}}_{2}^{w}\left(.\right)$ is an unbiased estimator for S_2_, characterized by a Gaussian asymptotic distribution with a variance that can be consistently estimated via bootstrap [2].

The first order tests are performed by considering the permutation distribution of the estimated distances TS_i_^1^ and TLS_i_^1^. When the cardinality $\prod_{j=1}^{k}{(m}_{j}+1)$ of the permutations is large, due to the large number of strata and to their size, it is worthwhile approximating the permutation distribution of the q test statistics with a Monte Carlo strategy that considers B random samples from the population of all possible permutations. Each of the B permutation samples is obtained by assigning, within each observed stratum, one subject to the experimental treatment and the remaining m_j_ to the standard treatment. The q first order tests are then calculated on each permuted sample and, at each time point, the partial p-values λ_i_’s for the q observed TS_i_^1^ or TLS_i_^1^ (i=1,…,q) are obtained from the empirical permutation distribution. Starting from the q permutation distributions, the p-values are also calculated for the first order test statistics of each permuted sample.

In order to test the global null hypothesis H_0_, the partial tests T_i_^1^ are subsequently combined in a unidimensional second-order test statistic T^2^. This combination has to be done nonparametrically, since the q univariate tests are dependent, and it is applied to the p-values λ_i_, which are permutationally equivalent to the partial tests [4]. Two alternative functions proposed by Fisher and Tippett, respectively, are considered, which can be applied on p-values either from TS_i_^1^ or TLS_i_^1^ as follows:

$$T_{F}^{2}=-2\sum_{i=1}^{q}{\log(\lambda }_{i})$$

$$T_{T}^{2}=\max_{1\leq i\leq q}\;{(1-\lambda }_{i})$$

As the Tippett combinig function can be written as $\underset{\;1\leq i\leq q}{1-\min}\;\lambda_{i}$, it has a monotonic relationship with the well known function $\min_{\;1\leq i\leq q}\;\lambda_{i}$ used by Westfall and Young [13] in their resampling approach to multiple testing.

While the q p-values λ_i_ are obtained from the marginal permutation distribution of the first order tests, the p-value of the global test is derived from the permutation distribution of the second order test, which is related to the q-variate distribution in the first step. Note that, no further permutations are needed at this stage, because we use, for the global test, the results on the p-values obtained from each permuted sample that define the permutation distributions of the first order tests [4].

In the survival setting the definition of the time points (t_1_,…,t_i_,…,t_q_) involved in the comparison may be relevant. We explored different strategies: i) fixed time points, ii) time points identified by percentiles of the overall event distribution and iii) all the observed event times included between the 10^th^ and the 90^th^ percentiles of the overall event distribution.

## Results

### Simulations

The performance of these tests in the finite sample situation are evaluated through simulations. The size and the power are calculated for different alternatives under proportional hazards and for three different scenarios of non-proportionality (e.g. survival curves showing both an early and a late difference or crossing each other). The behavior of the proposed tests is also explored in the presence of different number of strata (k=30, 50, 100) and of different degree of strata heterogeneity (no or low strata effect). The dimension of each stratum is defined by one case plus the number of controls generated by a Poisson (μ=4)+1 (1 is added in order to guarantee at least 1 control in each stratum).

**Figure 1:**
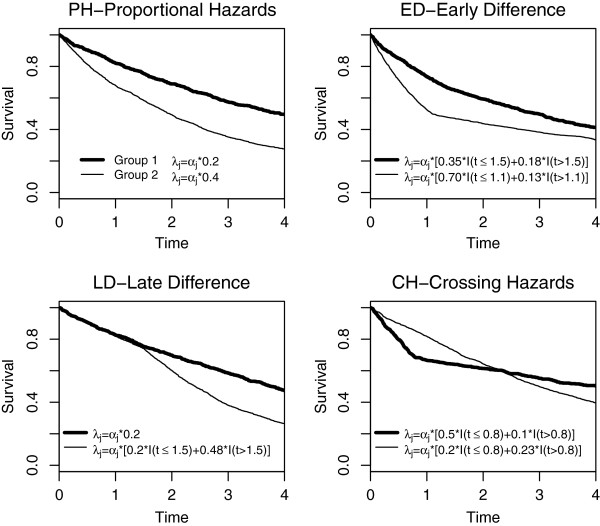
**Marginal survival curves under H**_**1**_**in the four scenarios considered in the simulation study.** For each scenario, we reported the stratum hazard functions used for data generation.

In the simulations under H_0_, the case and the matched controls share the same conditional piecewise exponential hazard function: λ_j_(t|α_j_,b_j_)= α_j_[b_1_I(t≤L)+ b_2_I(t>L)]. The randomness of λ_j_ is determined by α_j_~G[1/w,w], where G(a,b) is a Gamma distribution with mean ab and variance ab^2^, and the parameter w indicates the level of heterogeneity across strata (w=0, 0.22; specifically, for w=0, no strata effect is assumed as α_j_=1 with probability 1). Different scenarios are obtained by giving specific values to the landmark L, to b_1_ and b_2_. Under the alternative hypothesis, the hazard function for cases was decreased in order to define a survival which differs from that of controls, as specified in Figure 1. Censoring was uniform over 3–6 in order to have approximately 28–38 percent censoring, on average. For all the configurations studied, the results are based on 1000 samples and each of them uses B=2000 Monte Carlo permutations in order to define the empirical permutation distributions.

Definitions of the q time points involved in the comparison are as follows: i) 8 or 4 fixed equi-spaced times from 0.5-1 to 4, ii) 9 time points identified by the 10^th^, 20^th^,., 90^th^ percentiles of the overall event distribution, and iii) all the observed failure times between the 10^th^ and the 90^th^ percentiles of the overall event distribution.

The empirical level of the tests is shown in Tables 1.A (Fisher combining function) and 1.B (Tippett function), while the power in detecting a difference in survival is reported in Table 2.A-B, accordingly.

**Table 1:** **A-B - Simulation results under H**_**0**_**of the multivariate permutation (two-sided) Fisher (A) and Tippett (B) tests**

Scenario*	**Strata effect (w)**	**N. of strata (k)**	**TS**_**i**_^**1**^				**TLS**_**i**_^**1**^			
			**4 fixed**	**8 fixed**	**perc**	**all**	**4 fixed**	**8 fixed**	**perc**	**all**
**A) Fisher test**										
**PH**	0	30	0.047	0.046	0.053	0.046	0.046	0.052	0.055	0.043
	0	50	0.048	0.041	0.041	0.049	0.052	0.047	0.051	0.045
	0	100	0.044	0.045	0.043	0.044	0.047	0.038	0.045	0.045
	0.22	30	0.048	0.047	0.055	0.050	0.047	0.052	0.058	0.053
	0.22	50	0.050	0.047	0.048	0.050	0.051	0.051	0.053	0.048
	0.22	100	0.056	0.051	0.052	0.049	0.054	0.056	0.051	0.053
**ED**	0	30	0.057	0.056	0.062	0.062	0.065	0.061	0.055	0.054
	0	50	0.064	0.063	0.061	0.061	0.066	0.062	0.057	0.049
	0	100	0.052	0.050	0.053	0.056	0.050	0.049	0.049	0.053
	0.22	30	0.049	0.056	0.057	0.058	0.049	0.052	0.054	0.050
	0.22	50	0.038	0.040	0.043	0.042	0.037	0.039	0.044	0.048
	0.22	100	0.048	0.049	0.045	0.041	0.049	0.052	0.041	0.042
**LD**	0	30	0.047	0.047	0.051	0.055	0.046	0.050	0.056	0.053
	0	50	0.053	0.054	0.055	0.056	0.060	0.059	0.052	0.054
	0	100	0.046	0.048	0.053	0.050	0.049	0.050	0.050	0.049
	0.22	30	0.062	0.063	0.066	0.063	0.063	0.065	0.054	0.062
	0.22	50	0.059	0.057	0.056	0.057	0.054	0.054	0.056	0.054
	0.22	100	0.054	0.054	0.050	0.050	0.055	0.055	0.048	0.050
**CH**	0	30	0.052	0.049	0.050	0.052	0.059	0.051	0.052	0.057
	0	50	0.051	0.050	0.052	0.048	0.051	0.047	0.053	0.055
	0	100	0.049	0.046	0.038	0.037	0.050	0.051	0.043	0.047
	0.22	30	0.050	0.044	0.053	0.054	0.053	0.049	0.055	0.053
	0.22	50	0.043	0.040	0.040	0.038	0.044	0.041	0.049	0.048
	0.22	100	0.054	0.060	0.063	0.057	0.061	0.059	0.052	0.055
**B) Tippett test**										
**PH**	0	30	0.042	0.054	0.053	0.061	0.043	0.067	0.056	0.059
	0	50	0.041	0.048	0.043	0.047	0.050	0.056	0.053	0.049
	0	100	0.049	0.051	0.058	0.051	0.047	0.050	0.051	0.053
	0.22	30	0.057	0.059	0.058	0.065	0.060	0.069	0.053	0.071
	0.22	50	0.047	0.051	0.058	0.064	0.047	0.052	0.055	0.057
	0.22	100	0.051	0.052	0.056	0.059	0.055	0.058	0.050	0.054
**ED**	0	30	0.056	0.055	0.059	0.065	0.056	0.060	0.048	0.063
	0	50	0.064	0.069	0.066	0.070	0.063	0.065	0.055	0.054
	0	100	0.045	0.051	0.060	0.056	0.044	0.053	0.053	0.046
	0.22	30	0.053	0.062	0.059	0.066	0.056	0.054	0.062	0.064
	0.22	50	0.038	0.043	0.043	0.049	0.046	0.041	0.053	0.053
	0.22	100	0.042	0.049	0.038	0.047	0.045	0.053	0.047	0.052
**LD**	0	30	0.048	0.046	0.056	0.069	0.044	0.063	0.058	0.065
	0	50	0.044	0.051	0.055	0.049	0.050	0.061	0.052	0.059
	0	100	0.047	0.053	0.055	0.060	0.049	0.049	0.054	0.052
	0.22	30	0.061	0.058	0.069	0.066	0.062	0.065	0.056	0.056
	0.22	50	0.054	0.055	0.060	0.063	0.052	0.056	0.056	0.061
	0.22	100	0.057	0.055	0.057	0.050	0.062	0.064	0.061	0.057
**CH**	0	30	0.053	0.047	0.056	0.060	0.053	0.047	0.053	0.055
	0	50	0.054	0.049	0.045	0.054	0.051	0.046	0.047	0.063
	0	100	0.041	0.048	0.041	0.047	0.042	0.048	0.051	0.059
	0.22	30	0.059	0.047	0.054	0.066	0.054	0.055	0.067	0.062
	0.22	50	0.046	0.042	0.042	0.048	0.048	0.046	0.061	0.060
	0.22	100	0.061	0.062	0.063	0.066	0.061	0.065	0.055	0.054

* PH=Proportional Hazards, ED=Early Difference, LD=Late Difference, CH=Crossing Hazards.

The simulations are based on first order statistics TS_i_^1^ or TLS_i_^1^ under different scenarios and for different choices of time points (4 or 8 fixed or 9 points at 10-90^th^ percentiles (perc) or 80% of all the observed event times (all)).

**Table 2:** **A-B - Simulation results under H**_**1**_**of the multivariate permutation (two-sided) Fisher (A) and Tippett (B) tests**

Scenario*	**Strata effect (w)**	**N.of Strata(k)**	**TS**_**i**_^**1**^				**TLS**_**i**_^**1**^			
			**4 fixed**	**8 fixed**	**perc**	**all**	**4 fixed**	**8 fixed**	**perc**	**all**
**A**) **Fisher test**										
**PH**	0	30	**0.734**	**0.723**	0.627	0.592	**0.744**	**0.737**	0.673	0.651
	0	50	**0.911**	**0.906**	0.854	0.822	**0.909**	**0.906**	0.871	0.847
	0	100	**0.998**	**0.996**	0.993	0.968	**0.997**	**0.996**	0.993	0.978
	0.22	30	**0.692**	**0.691**	0.606	0.578	**0.706**	**0.720**	0.658	0.638
	0.22	50	**0.882**	**0.873**	0.816	0.795	**0.888**	**0.887**	0.839	0.831
	0.22	100	**0.995**	**0.995**	0.983	0.978	**0.997**	**0.995**	0.985	0.981
**ED**	0	30	0.274	0.274	0.414	0.432	0.302	0.297	**0.514**	**0**.**534**
	0	50	0.406	0.408	0.622	0.641	0.443	0.452	**0.682**	**0.699**
	0	100	0.730	0.746	0.916	0.927	0.760	0.770	**0.939**	**0.937**
	0.22	30	0.240	0.252	0.376	0.409	0.267	0.285	**0.470**	**0.488**
	0.22	50	0.387	0.252	0.616	0.632	0.423	0.285	**0.671**	**0**.**682**
	0.22	100	0.710	0.727	0.894	0.902	0.739	0.753	**0.924**	**0.929**
**LD**	0	30	**0.425**	**0.408**	0.278	0.247	**0**.**408**	**0.400**	0.298	0.266
	0	50	**0.674**	**0.642**	0.464	0.392	**0.648**	**0.627**	0.470	0.406
	0	100	**0.949**	**0.937**	0.802	0.749	**0.940**	**0.928**	0.796	0.691
	0.22	30	**0.399**	**0**.**369**	0.243	0.209	**0.387**	**0.373**	0.273	0.245
	0.22	50	**0.626**	**0.590**	0.414	0.359	**0.620**	**0.587**	0.440	0.384
	0.22	100	**0.910**	**0.890**	0.740	0.712	**0.908**	**0.888**	0.751	0.725
**CH**	0	30	0.144	0.165	0.285	0.272	0.079	0.078	0.115	0.099
	0	50	0.291	0.321	0.488	0.461	0.162	0.148	0.229	0.207
	0	100	0.747	0.799	0.897	0.881	0.560	0.596	0.746	0.729
	0.22	30	0.149	0.181	0.273	0.248	0.087	0.089	0.111	0.105
	0.22	50	0.292	0.319	0.477	0.419	0.178	0.161	0.237	0.207
	0.22	100	0.736	0.794	0.878	0.844	0.575	0.589	0.744	0.668
**B**) **Tippett test**										
**PH**	0	30	0.685	0.666	0.586	0.605	0.676	0.654	0.674	0.677
	0	50	0.874	0.866	0.828	0.807	0.878	0.869	0.856	0.875
	0	100	0.996	0.995	0.991	0.979	0.996	0.994	0.992	0.996
	0.22	30	0.646	0.632	0.567	0.572	0.657	0.663	0.659	0.684
	0.22	50	0.834	0.826	0.768	0.766	0.850	0.831	0.816	0.814
	0.22	100	0.992	0.990	0.976	0.975	0.993	0.990	0.982	0.979
**ED**	0	30	0.393	0.369	0.351	0.384	0.458	0.458	**0.531**	**0.596**
	0	50	0.615	0.571	0.579	0.597	0.661	0.646	**0.679**	**0.708**
	0	100	0.906	0.910	0.904	0.914	0.920	0.924	**0.937**	**0.942**
	0.22	30	0.369	0.343	0.347	0.365	0.429	0.422	**0.501**	**0.544**
	0.22	50	0.597	0.343	0.560	0.589	0.652	0.422	**0**.**676**	**0.699**
	0.22	100	0.887	0.877	0.896	0.897	0.910	0.901	**0**.**923**	**0.925**
**LD**	0	30	**0.472**	**0.458**	0.392	0.383	**0.438**	**0.442**	0.398	0.400
	0	50	**0**.**730**	**0.718**	0.644	0.632	**0.688**	**0.683**	0.634	0.615
	0	100	**0**.**963**	**0**.**957**	0.926	0.914	**0.956**	**0.950**	0.920	0.918
	0.22	30	**0.425**	**0**.**397**	0.342	0.318	**0.411**	**0.393**	0.363	0.361
	0.22	50	**0.660**	**0.650**	0.572	0.567	**0.658**	**0.634**	0.584	0.566
	0.22	100	**0.930**	**0.924**	0.871	0.864	**0.927**	**0.920**	0.874	0.858
**CH**	0	30	0.356	0.418	**0.475**	**0**.**521**	0.183	0.155	0.191	0.163
	0	50	0.584	0.636	**0.700**	**0.729**	0.380	0.335	0.374	0.347
	0	100	0.920	0.931	**0.951**	**0.971**	0.854	0.842	0.851	0.848
	0.22	30	0.335	0.399	**0.433**	**0.484**	0.176	0.165	0.185	0.163
	0.22	50	0.560	0.626	**0.663**	**0.722**	0.408	0.358	0.385	0.348
	0.22	100	0.887	0.921	**0.953**	**0.963**	0.830	0.806	0.850	0.836

* PH=Proportional Hazards, ED=Early Difference, LD=Late Difference, CH=Crossing Hazards

The simulations are based on first order statistics TS_i_^1^ or TLS_i_^1^ under different scenarios and for different choices of time points (4 or 8 fixed or 9 points at 10-90^th^ percentiles (perc) or 80% of all the observed event times (all)). In the body of the table, values in bold identify the strongest results.

As expected, the performance of the test Under H_0_ is generally good in all settings. In particular, with a small number of strata, the test based on the survival distance behaves very well. When the complementary log-log transform is considered, the permutation distribution at early time points, such as the 10^th^ percentile, may be very irregular because this transformation magnifies survival values near one. In order to have a good behavior even with small number of strata, it is sufficient to start from a higher percentile such as the 20^th^ (data not shown).

Under H_1_, as expected, power increases with increasing number of strata under every scenario and the test has a good behavior also in the situation of crossing hazards. No major gain in power is achieved by increasing the number of fixed time points from 4 to 8 and similar results are obtained when points are fixed at percentiles of the event distribution or take 80% of all observed event times. The fixed time point strategy seems generally more satisfactory for the proportional hazards and the late difference scenarios. A clear advantage in the use of the complementary log-log transform is seen only in the early difference scenario; conversely the survival difference seems to offer an advantage in the crossing hazards setting. Again, by comparing Table 2.A and 2.B, where the best performers for each scenario are in bold, the performance under the Fisher and Tippet functions is very similar, except for an advantage of Tippett in the crossing hazards scenario.

Results on the comparison of our permutation tests with other approaches are reported in Table 3. The stratified log-rank test, the modified log-rank test by Schoenfeld-Tsiatis and the robust test from the Cox model including only the covariate for treatment have size close to the nominal level. The multivariate permutation tests perform better than the latter tests in terms of power in the crossing hazards and early difference scenarios, while they have a similar behavior in the presence of proportional hazards.

**Table 3:** **Simulation results under H**_**0**_**and H**_**1**_**of the tests used for comparative purposes**

Scenario*	**Strata effect(w)**	**N.of strata(k)**	**Under H**_**0**_			**Under H**_**1**_		
			**(1)**	**(2)**	**(3)**	**(1)**	**(2)**	**(3)**
**PH**	0	30	0.042	0.043	0.049	0.660	**0.734**	**0.770**
	0	50	0.042	0.043	0.051	0.867	**0.923**	**0.938**
	0	100	0.054	0.050	0.060	0.995	**0.999**	**1.000**
	0.22	30	0.044	0.052	0.053	0.635	**0.731**	**0.726**
	0.22	50	0.057	0.051	0.058	0.827	**0.898**	**0.903**
	0.22	100	0.056	0.054	0.053	0.986	**0.997**	**0.996**
**ED**	0	30	0.053	0.061	0.064	0.207	0.189	0.216
	0	50	0.061	0.067	0.071	0.317	0.262	0.310
	0	100	0.049	0.052	0.061	0.620	0.531	0.571
	0.22	30	0.046	0.051	0.056	0.204	0.169	0.166
	0.22	50	0.037	0.044	0.045	0.292	0.237	0.253
	0.22	100	0.051	0.048	0.053	0.596	0.501	0.510
**LD**	0	30	0.042	0.046	0.054	0.384	0.473	0.520
	0	50	0.053	0.059	0.060	0.600	0.731	0.775
	0	100	0.052	0.052	0.118	0.863	0.965	0.971
	0.22	30	0.049	0.065	0.070	0.338	0.454	0.470
	0.22	50	0.046	0.051	0.051	0.553	0.707	0.705
	0.22	100	0.051	0.056	0.051	0.816	0.949	0.947
**CH**	0	30	0.062	0.070	0.076	0.076	0.078	0.082
	0	50	0.045	0.045	0.046	0.087	0.119	0.119
	0	100	0.041	0.045	0.048	0.095	0.174	0.662
	0.22	30	0.046	0.043	0.049	0.079	0.091	0.083
	0.22	50	0.040	0.042	0.039	0.101	0.142	0.138
	0.22	100	0.063	0.056	0.064	0.116	0.233	0.233

* PH=Proportional Hazards, ED=Early Difference, LD=Late Difference, CH=Crossing Hazards.

The outcomes of the stratified log-rank test (1), the modified log-rank by Schoenfeld and Tsiatis for highly stratified data (2) and the Cox model with sandwich variance that accounts for stratified data with possibly correlated failure times (3) are reported. In the body of the table, values in bold identify the strongest results.

### Application

**Figure 2:**
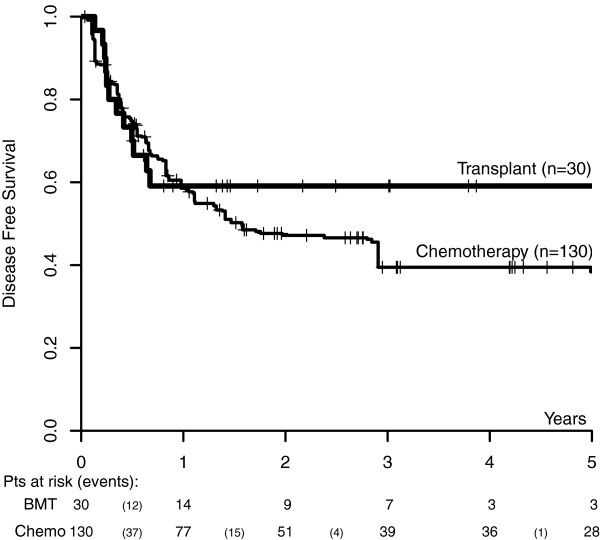
**Disease free survival curves of transplanted patients (n=30) and matched chemotherapy controls (n=130).** The estimates are calculated according to standard Kaplan-Meier and to the weighted version of Kaplan-Meier, respectively.

The clinical context that motivated this work is a retrospective multicentre study on 427 children enrolled between 1985 and 1994 in high-risk protocols for the treatment of ALL, where 30 received an allogeneic bone-marrow transplant after achieving first remission. In this setting, where transplant was considered as an experimental treatment to be administered only to selected patients, we used matching to define the appropriate group of children treated with chemotherapy alone to be used for an unbiased comparison. The matching procedure was based on 6 factors (centre, front-line treatment protocol, white blood cell count and age at diagnosis, immunophenotype and waiting time to transplant). For each of the 30 BMT cases, at least one non transplanted matched control was found, with a maximum of 17 and a median of 3, for a total of 130 controls. The Disease Free Survival (DFS) curves of the two groups depicted in Figure 2 were estimated using the standard and the weighted version of Kaplan-Meier for the transplant and the chemotherapy group, respectively. This plot indicates a long term advantage of transplant over chemotherapy after 1 year from transplant, with a DFS at 3 years of 59.1% (Greenwood s.e. 9.1) and 39.5% (bootstrap s.e. 7.7), respectively.

A local two-sided permutation test was originally used on the estimated DFS difference of 19.6% at the time point of 3 years, considered a meaningful time point according to clinical experience (p-value=0.112). At 3 years, 7 cases and 39 controls were still at risk and most of the overall number of events (97%) had already occurred, as shown by the event distribution below Figure 2. We have now applied the multivariate permutation approach in order to compare the two DFS curves with a global test. The Fisher and the Tippett tests are performed based both on the direct estimates of survival and on its complementary log-log transformation, according to different choices of time points involved in the comparison. Based on previous studies and knowledge on the treatment implications on toxicity and survival, the most likely alternative to the null is a non proportional setting with an early crossing and late differences in hazards. The result in term of significance (p-value=0.199) points to the Tippett test on log-survival involving 4 points (at 1, 2, 3 and 4 years from the origin). Other tests on 4 fixed time points, starting from 0.5 or 1 year (and not necessarily equispaced), are applied, and results are in keeping with the observation that curves start diverging at 1 year. The tests involving the percentiles and all the event times within the 10^th^ and 90^th^ percentile, give p-values ranging from 0.486 to 0.829 (Table 4).

**Table 4:** **Results of different multivariate permutation** (**two**-**sided**) **tests applied to the application**

**Time points strategy**	**N.of time points(years)**	**Global p-value**			
		**TS**_**F**_^**2**^	**TLS**_**F**_^**2**^	**TS**_**T**_^**2**^	**TLS**_**T**_^**2**^
**fixed**	4 (0.5,1,2,3)	0.387	0.375	0.263	0.245
**fixed**	4 (1,1.5,2,3)	0.348	0.338	0.225	0.212
**fixed**	4 (1,2,3,4)	0.232	0.221	0.213	0.199
**perc**	9 (0.16-1.74)*	0.665	0.675	0.515	0.486
**all**	52 (0.19-1.71)*	0.799	0.829	0.630	0.579

* min and max time points.

Results of the comparison of Disease Free Survival (DFS) curves of transplanted patients (n=30) and matched chemotherapy controls (n=130) are reported. Global p-values are shown for Fisher and Tippett second order statistics based on the distance of DFS curves (TS_i_^1^) or their transformation (TLS_i_^1^) and for different choices of the time points (fixed or points at 10-90^th^ percentiles (perc) or 80% of all the observed event times (all)).

Due to the relatively small sample size, the power is likely not adequate to detect the advantage given by transplantation, and this holds true even if we are applying a more appropriate test with regard to standard methods (stratified log-rank test, p-values=0.966; Cox model with robust standard error, p-values=0.915).

## Discussion

This paper provides a global test for the comparison of survival curves in the context of highly stratified data produced by matching on a non-fixed proportion, by extending the multivariate permutation approach proposed by Pesarin [4]. The nonparametric combination of partial tests is able to capture their underlying dependence without assumptions and to control the level of significance of the global test, avoiding the issue of multiplicity. In principle, the proposed tests can be viewed as a weighted combination of an infinite number of time points, giving weight 1 to those involved in the comparison, and weight 0 otherwise. Yet, in survival analysis, the choice of the time points where partial tests are performed is a crucial issue. Our approach carefully considers this aspect by evaluating three different convenient strategies among the many possible. The most simple is the one that identifies a number of fixed time points. This is appealing for applications where the researcher has reasons for evaluating certain times that are relevant for the phenomenon. Importantly, given the degree of arbitrariness involved, the choice of the time points should be carefully done, possibly a-priori, in a time window where more information is expected, given the experimental setting and the follow-up. Typically, in time points near the boundaries, i.e. at the very beginning or on the tail of the survival curves, where few events are generally observed, the permutation distribution of the partial tests may be irregular. Following this consideration, alternative non subjective definitions of time points are based on the observed distribution of events, either with a limited number defined by percentiles, or on the whole set of event times within the 10^th^ up to the 90^th^ percentile.

As expected, the extended simulation study shows good performances of the proposed test in terms of alpha coverage. Under the alternative, apparently a slight advantage is obtained considering all event times for partial tests in the crossing hazard and early difference scenarios. We could generally recommend the Tippett transform with some caution on small number of strata where, to achieve a regular behaviour, it is however sufficient to start from a percentile higher than the 10^th^. The Fisher test is very similar, slightly better under proportional hazards, but worst in the crossing hazards setting. Differently from what found in a non permutation test by Klein et al. [12], we do not observe a general superiority of the complementary log-log transform of survival on survival, as distance measure, except in the early distance scenario. Actually, the Tippett transform behaves well using the survival distance even with small number of strata. While a standardized survival difference could have been considered, the expected gain in performance would not counterbalance the heavy additional computational effort needed. This because the standard error of the difference is based on a bootstrap estimate that should account for the complex form of dependence possibly induced by matching, that cannot be easily specified.

In the context of non matched data (or no strata effect), the proposed multivariate permutation test maintains a good performance for survival data comparison. Indeed, this approach could be useful even in non matched data in the presence of departures from the proportional hazards assumption, especially crossing hazards, where an overall test is still matter of development.

Conditions for the applicability of this approach are very mild. The appropriate application of the multivariate permutation testing approach relies on the assumptions on censoring being independent from the failure process and treatment within strata. While this could be considered a weakness, it is indeed the usual assumption which applies in survival when a correct study planning is adopted. The test relies also on the assumption of exchangeability under the null. In our context, where permutations are within strata, this latter assumption is reasonable due to matching. Finally, the test can easily be extended to more than one subject of the experimental arm in each stratum, modifying the weighting scheme of the Kaplan-Meier estimator of survival.

Other permutation tests were not suitable for extension to this context. Specifically, the proposals of both Pesarin-Salmaso [5] and of Heller-Venkatraman [8] do not account for stratified and possibly correlated data; the approach of Sun and Sherman [9] needs relatively large strata of independent observations, and the versatile test of Shih and Fay [10], while dealing with highly stratified matched data, behaves similarly to the log-rank test or its stratified version, depending on the level of inter-strata heterogeneity.

## Conclusions

In conclusion, our approach is different from other permutation tests proposed for survival analysis [8-10], as it relies on the multivariate permutation approach originally introduced by Pesarin [4]. It is recommended in the highly stratified context of matched data where it is proven by simulations to be at least equal or superior to the stratified log-rank test, the modified log-rank test for highly stratified data and a Cox model with robust variance estimate. Preference to this test should be definitely given when the proportional hazards assumption does not hold, even in non matched data. As a general indication, we would suggest the application on survival differences at fixed time points for the first order test and the Tippett transform for the global combining function. This test requires some computational effort, but it is, in our experience, quite feasible with ad-hoc routines (one in fortran, as well as a function in R, can be provided by the authors upon request).

## Abbreviations

ALL: Acute lymphoblastic leukaemia; BMT: Bone Marrow Transplantation; PH: Proportional Hazards; ED: Early Difference; LD: Late Difference; CH: Crossing Hazards.

## Competing interests

The authors declare that they have no competing interests.

## Authors’ contributions

SG performed the simulation studies and drafted the manuscript. MGV supervised the analyses and contributed to the manuscript. All authors read and approved the final manuscript.

## Pre-publication history

The pre-publication history for this paper can be accessed here:

http://www.biomedcentral.com/1471-2288/13/16/prepub
